# CD63 Promotes Hemocyte-Mediated Phagocytosis in the Clam,* Paphia undulata*

**DOI:** 10.1155/2016/7893490

**Published:** 2016-10-27

**Authors:** Mingjia Yu, Shanjun Yang, Hongxia Sun, Qiang Xia

**Affiliations:** ^1^Engineering Research Center of Marine Biological Resource Comprehensive Utilization, Third Institute of Oceanography, State Oceanic Administration, Xiamen 361005, China; ^2^Department of Immunology and Pathogen Biology, Zunyi Medical College, Zhuhai Campus, Zhuhai 519041, China

## Abstract

As one of the surface membrane proteins of tetraspanin family, CD63 plays a crucial role in cellular trafficking and endocytosis, which also is associated with activation of a wide variety of immune cells. Here, the homolog of CD63 was characterized from one marine mollusk,* Paphia undulata*, which is designated as Pu-CD63. The complete cDNA of Pu-CD63 is 1,738 bp in length with an open reading frame (ORF) of 849 bp, encoding a 282 amino acid protein with four putative hydrophobic transmembrane helixes. Bioinformatic analysis revealed that Pu-CD63 contains one putative YXXØ consensus motif of “110-YVII-113” and one N-glycosylation site “155-NGT-157” within the large extracellular loop (LEL) region, supporting its conserved function in plasma membrane and endosomal/lysosomal trafficking. Moreover, temporal expression profile analysis demonstrates a drastic induction in the expression of CD63 in hemocytes after pathogenic challenge with either* V. parahaemolyticus *or* V. alginolyticus*. By performing dsRNA-mediate RNAi knockdowns of CD63, a dramatic reduction in hemocytes phagocytic activity to pathogenic* Vibrio *is recorded by flow cytometry, revealing the definite role of Pu-CD63 in promoting hemocyte-mediated phagocytosis. Therefore, our work has greatly enhanced our understanding about primitive character of innate immunity in marine mollusk.

## 1. Introduction

CD63 is a member of the tetraspanin family of cell surface associated membrane proteins characterized by presence of four hydrophobic domains [1]. Originally found to be present on the cell surface of activated blood platelets in humans, CD63 was later shown to function as the activation mark itself [2]. At the cell surface, CD63 is known to be endocytosed via a clathrin-dependent pathway [3]. Recent evidences, however, suggest the involvement of additional pathways in the process [4]. In addition to the plasma membrane, CD63 is highly expressed in the endosome and lysosome of immune cells including antigen presenting cells, T-lymphocytes, and neutrophils [5–7]. In the macrophages and dendritic cells, CD63 can chaperone MHC molecules through the endosomal pathway to present antigens and initiate immune response [8], whereas, in the T-lymphocytes, CD63 is linked to the proper trafficking of cell surface associated chemokine receptor (CXCR) and its mutation leads to mistargeting of CXCR to late endosomes/lysosomes [3, 7]. This also indicates the crucial role CD63 plays in cellular trafficking. Additionally, CD63 has also been associated with a wide variety of cancer cells, where it plays a role in cell activation, motility, differentiation, and tumor invasion making this protein an important research topic in vertebrates [9].

In invertebrates, hemocyte-mediated phagocytosis is a specific form of endocytosis which involves engulfment of pathogenic bacteria. This process plays a central role in efficiently eliminating the invading pathogens aiding in host defense [10]. The internalized vacuole harboring the pathogen, called phagosomes, then undergoes maturation through lysosomal fusion resulting in acidification and thorough digestion of engulfed bacteria [11, 12]. We believe that CD63 protein is involved in the process of hemocyte-mediated phagocytosis. Interestingly, immunofluorescence studies have demonstrated that CD63 is highly expressed in the granular and vesicular structures of mollusk hemocytes, supporting our hypothesis about its possible role in phagocytosis [13].

*P. undulata* is one of the most important economic marine mollusks in the South China Sea and Gulf of Thailand [14]. Recently, it has been suffering from severe pathogenic infections resulting in collapsing natural clam populations inflicting great losses to the aquaculture industry [15, 16]. Therefore, understanding the molecular mechanism of innate immunity in the clam* P. undulata* is of paramount importance. In this study we have investigated the possible role of* P. undulata* CD63 protein in promoting hemocyte-mediated phagocytosis against pathogenic challenge. In the process we have cloned and characterized the full-length cDNA of CD63 from the clam. Our results provide insights into the basic defense mechanisms of the marine bivalve.

## 2. Materials and Methods 

### 2.1. Experimental Animals, Bacterial Challenge, and Hemocytes Collection

Healthy clams were obtained from Xiamen, Fujian Province, China, and were maintained in tanks at 18°C–22°C with recirculating seawater for two week, prior to the bacterial infection. The clams were challenged with injection of 100 *μ*L* Vibrio parahaemolyticus* or* Vibrio alginolyticus* at a concentration of 1 × 10^7^ cells/mL. The controls were injected with equal volume of PBS instead. At 24 h after injection, hemolymph was withdrawn from the adductor muscle of clams with a 0.5 mm diameter (25 G) disposable needle and immediately centrifuged (700 ×g for 10 min at 4°C) to separate the hemocytes cells from plasma. For each treatment, approximately 20 individuals were pooled and stored at −80°C for RNA isolation [17].

### 2.2. Gene Cloning and Bioinformatic Analysis

CD63 partial fragments were obtained from the* de novo* transcriptome of* P. undulate* hemocytes. A set of gene specific primers (Table 1) were used to amplify 5′ and 3′ cDNA ends using GeneRacer™ Kit (Life Technologies, USA). The RACE products were cloned into the pCR-TOPO Vector (Life Technologies, USA) and were sequenced with ABI Prism 3730 DNA sequencer (PerkinElmer, Wellesley, MA, USA). Finally, the full-length cDNA sequence of* P. undulata* CD63 was constructed by combining the 3′- and 5′-end sequences.

The full-length CD63 sequence was analyzed using the NCBI BLAST algorithm (http://www.ncbi.nlm.nih.gov/blast) and the Expert Protein Analysis System (http://www.expasy.org/). Protein domain prediction was performed by SMART (http://smart.embl-heidelberg.de/) web tool and the presence of transmembrane structure was analyzed using TMHMM (http://www.cbs.dtu.dk/services/TMHMM/). Multiple sequence alignments of CD63 amino acid sequences were performed using the ClustalX version 1.81 and the phylogenetic trees were generated by the MEGA 5.0 package utilizing neighbor-joining method.

### 2.3. Quantitative Real-Time PCR

Total RNA was extracted from hemocytes with TRIzol Reagent (Invitrogen, USA) according to the manufacturer's directions. The integrity of the RNA was assessed by agarose gel electrophoresis. cDNA was reverse-transcribed using the PrimeScript™ RT Reagent Kit Ver.2.0 (TaKaRa, Japan) from 1 *μ*g of total RNA. The expression profile of CD63 in bacteria challenged hemocytes was measured by quantitative real-time PCR (qPCR). The qPCRs were carried out in a 20 *μ*L reaction system containing 10 *μ*L of 2x Master Mix (Roche, USA), 0.4 *μ*L of each of the forward and reverse primers (10 mM), 1 *μ*L of diluted cDNA (1 : 10), and 8.2 *μ*L of PCR-grade water. Glyceraldehyde 3-phosphate dehydrogenase (GAPDH) was used as the reference gene and additional negative controls were also used to test for genomic DNA contaminations. qPCRs were performed on the Light Cycler 480 platform (Roche). The specificity of the PCR amplifications was accessed by analyzing the melting curves at the end of the reaction. All data were generated by the Rotor-gene version 6-0-22 software and the fold changes were calculated using the 2-Ct method [18]. All experiments were done in triplicate.

### 2.4. RNA Interference* In Vivo*

Appropriate fragments of CD63 and control Luciferase (obtained from pGL3-vector) were PCR-amplified with T7 promoter linked primers (Table 1). The products were used as templates for dsRNA synthesis by* in vitro *transcription using MEGAscript T7 Transcription Kit (Life Technologies, USA). The RNA integrity was examined by gel electrophoresis and the concentration was quantified by spectrophotometers of NanoDrop 2000C (Thermo Scientific).* In vivo* gene knockdowns were performed by injecting 20 *μ*g respective dsRNA into the adductor of the clam. RNAi efficiency was determined by qPCR 72 h after dsRNA injection.

### 2.5. Phagocytosis and FACS Analysis

The phagocytic activity of hemocytes was measured by flow cytometry (BD FACSCalibur) as described previously [19]. Briefly, hemolymph was collected from normal and CD63 knockdown clams and fluorescent (FITC) conjugated* V. parahaemolyticus* and* V. alginolyticus *were incubated with the hemolymph. After 30 minutes of incubation, cell-associated fluorescence was quantified using flow cytometry at 530 nm (FL1) to assess phagocytic activity. Hemocytes cell populations were defined based on their size (FSC) and granularity (SSC) properties, and a total of 10,000 events were acquired for each sample. The mean fluorescent intensity in different populations was determined using the Cell Quest Software [19].

### 2.6. Statistical Analysis

The statistical differences between groups were analyzed by one-way ANOVA, followed by Dunnett test and Turkey multiple comparison test using the SPSS software package. For pairwise comparisons, Student's *t*-test was used instead. *P* values of less than 0.05 or 0.01 were used to indicate statistical significance. All data are represented as means ± S.D.

## 3. Results and Discussion 

**Figure 1:**
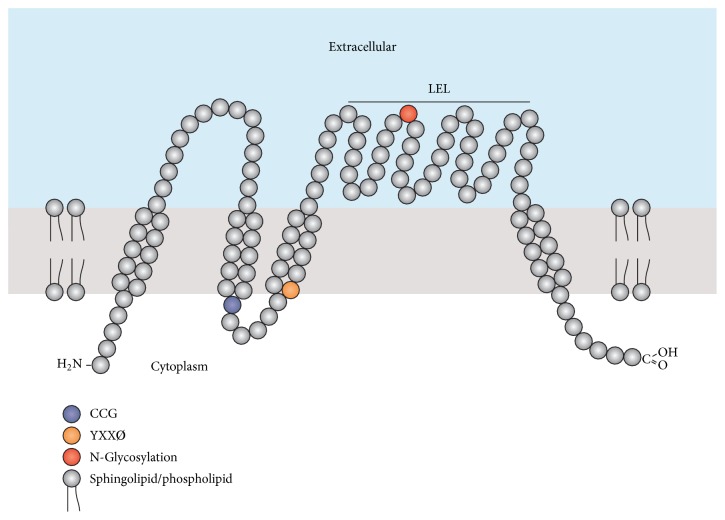
Transmembrane structure of Pu-CD63 protein.

In this study, the homolog of CD63 was identified and characterized from marine mollusk,* Paphia undulata*, henceforth designated as Pu-CD63. The complete cDNA of Pu-CD63 is 1,738 bp in length and includes an open reading frame (ORF) of 849 bp, a 5′-untranslated region (UTR) of 168 bp, and a 3′-UTR of 721 bp with a canonical polyadenylation signal (AATAAA) (Supplementary Figure 1, in Supplementary Material available online at http://dx.doi.org/10.1155/2016/7893490). This previously uncharacterized Pu-CD63 cDNA sequence has been deposited in GenBank under accession number KT596763. Bioinformatics analysis showed that the ORF encodes a 282-amino-acid protein with a calculated molecular mass of 32 kDa and a theoretical isoelectric point of 8.32. TMHMM analysis revealed that Pu-CD63 contains four putative hydrophobic transmembrane helixes (Figure 1), with the characteristic large extracellular loop (LEL) between the third and the fourth transmembrane domain [4, 20, 21].

CD63 proteins are majorly trafficked to either the plasma membrane in cell surface or the endosomes/lysosomes. Two characteristics motifs of CD63, YXXØ consensus motif and N-glycosylation sites, assist in this targeting. The YXXØ motif contains an essential tyrosine residue, 2 hydrophobic XX residues, and a bulky hydrophobic amino acid and plays a role in both endocytosis of CD63 from the plasma membrane and lysosomal targeting [22]. The sequence “110-YVII-113” is located within the third transmembrane helix and meets the criterion for YXXØ motif, implicating a possible role in Pu-CD63 trafficking. On the other hand, the other motif, N-linked glycosylation, was shown to be required exclusively for endosomal/lysosomal targeting of CD63 in mammals [23, 24]. Bioinformatic analysis also revealed that Pu-CD63 contains one putative N-glycosylation site “155-NGT-157” within the LEL region, suggesting its conserved function in endosomal/lysosomal trafficking. In addition to four transmembrane domains the pu-CD63 proteins feature several conserved amino acid residues including a highly conserved CCG motif, the two cysteine residues of which are required for essential disulphide bond formations in the second extracellular loop [25, 26].

Phylogenetic trees were constructed from sequence alignments of homologous CD63 proteins from different species by utilizing the neighbor-joining method (Supplementary Figure 2). In agreement with the traditional evolutionary groupings, Pu-CD63 was clustered together with its homolog from another mollusk species* Crassostrea gigas* into the mollusk clade.

**Figure 2:**
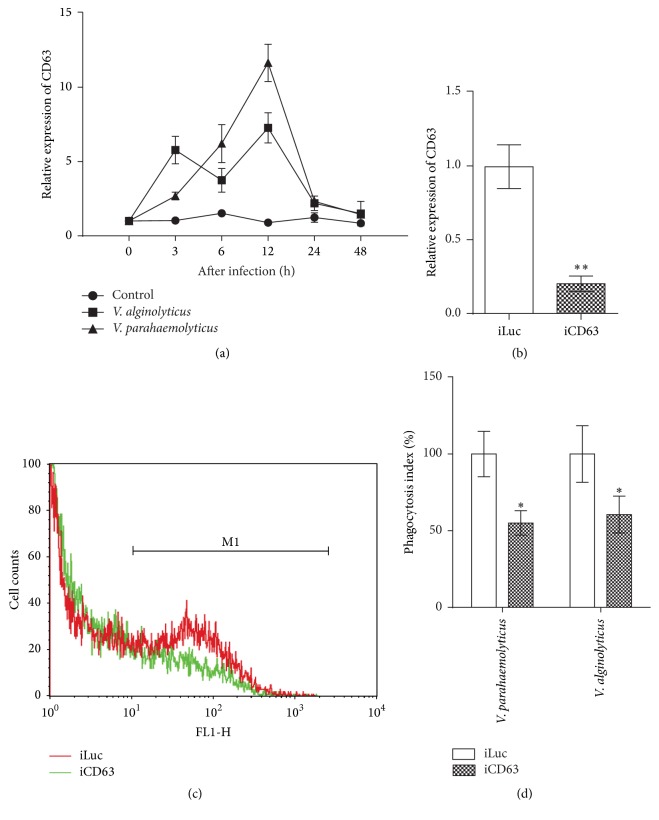
CD63 is involved in the hemocyte-mediated phagocytosis in clams. (a) mRNA profile of Pu-CD63 after bacterial infection; (b) RNAi mediated knockdown of CD63; (c), (d) phagocytic index in iPu-CD63 clams.  ^*∗*^Significant difference with *p* < 0.05,  ^*∗∗*^significant difference with *p* < 0.01.

To explore the possible involvement of Pu-CD63 in the immune response, its temporal expression profile was determined in the hemocytes after bacterial challenge, using qPCR. In comparison to the uninfected controls, Pu-CD63 transcript sharply increased to about 6-fold and 3-fold at 3 h after challenge with* V. parahaemolyticus *and* V. alginolyticus*, respectively (Figure 2(a)). Its expression peaked at 12 h after* V. parahaemolyticus *and* V. alginolyticus *challenge, with respective 12- and 7-fold change. 24–48 h after challenge, however, the transcript abundance of Pu-CD63 dropped to basal levels. These results indicate a clear induction in the expression of CD63 in mollusk hemocytes after pathogenic challenge and strongly support our hypothesis about an immunogenic role of Pu-CD63. A similar induction of the CD63-like gene was observed in the hemocytes of* Crassostrea ariakensis* 12 h after challenge with lipopolysaccharide (LPS) and polyinosinic:polycytidylic acid (poly I:C) [13], supporting our finding.

As the predominant cell-mediated defense mechanism for tackling the invading pathogens, phagocytosis plays a central role in the innate immune response of invertebrates, including mollusks. To examine the potential role of CD63 on the hemocyte-mediated phagocytosis in* P. undulata*, dsRNA-mediate RNAi knockdown of CD63 was performed* in vivo* by injection into the adductor muscle [27]. The knockdown efficiency of Pu-CD63 mRNA was assessed in the hemocytes via qPCR at 72 h after injection. The level of the Pu-CD63 mRNA decreased to about 20% of that of the controls (Figure 2(b)). With 70% inhibition set as the accepted threshold for efficient RNAi [28], our injection successfully knocked down the Pu-CD63 gene in clam hemocytes. We used these iPu-CD63 clams for further analysis.

The fluorescent (FITC) conjugated* V. parahaemolyticus* and* V. alginolyticus *were incubated with hemolymph to assess phagocytic activity of iPu-CD63 clams. A dramatic 78% and 33% reduction in hemocytes phagocytic activity was recorded in iPu-CD63 clams by flow cytometry, after incubation with* V. parahaemolyticus* and* V. alginolyticus*, respectively (Figures 2(c) and 2(d)). This result clearly demonstrated the role of CD63 proteins in hemocytes mediated phagocytosis in response to pathogenic challenge in the clam* P. undulata*.

Supporting our observation, there are reports that internalization of CD63 is accompanied with phagocytosis of* Saccharomyces cerevisiae* in human dendritic cells [29]. Although the exact mechanism of how CD63 promotes phagocytosis remains unclear, previous studies have demonstrated that it can serve as an adaptor protein linking different other proteins via direct/indirect interaction and facilitating the formation of endocytic machinery [30]. Apart from various cell surface receptors, CD63 has also been shown to associate with integrin proteins [31, 32]. Interestingly, in mollusks, blockage of integrin activity could significantly reduce the phagocytic ability of the hemocytes [10]. We believe that CD63 promotes hemocyte-mediated phagocytosis via altering the integrin recruitment in endocytic machinery, a hypothesis which needs to be tested in the future.

In summary, we have cloned and characterized the CD63 sequence from clam* P. undulata*, followed by its expression profiling in pathogen challenged hemocytes. A drastic induction in the Pu-CD63 mRNA was noted in the hemocytes after pathogen challenge. Moreover, using flow cytometry we showed a definite role of Pu-CD63 in promoting hemocyte-mediated phagocytosis. Our work has enhanced our understanding about innate immunity of economically important clam* P. undulata*.

## Supplementary Material

Supplementary Figure 1: The complete cDNA and deduced amino acid sequence of Pu-CD63.
Supplementary Figure 2: Phylogenetic tree of CD63.

## Figures and Tables

**Table 1:** 

Primer	Sequence (5′ → 3′)	Comment
GR5P	CGACTGGAGCACGAGGACACTGA	Adaptor of 5′ RACE
GR5NP	GGACACTGACATGGACTGAAGGAGTA	Adaptor of 5′ RACE
GR3P	GCTGTCAACGATACGCTACGTAACG	Adaptor of 3′ RACE
GR3NP	CGCTACGTAACGGCATGACAGTG	Adaptor of 3′ RACE
CD63-R1	GATGGTCAGAAACAGGCCAAAGAAA	5′ RACE primer of First PCR
CD63-F1	GCAGACACTTCCACAGCGACGACAG	3′ RACE primer of First PCR
CD63-R2	CCGAAGCGAATAAACAAGCCCAGTCCGAAA	5′ RACE primer of Nest PCR
CD63-F2	TCATCTGGGACGCCACCCTTGGAAGTAC	3′ RACE primer of Nest PCR
CD63-QF	TAGATAAGGGATGTTACGAC	Real-time PCR for Pu-CD63
CD63-QR	TTCATGCTACACAAAATTAC	Real-time PCR for Pu-CD63
GAPDH-QF	ACTCGGAAAGGAGACCACCTACCA	Real-time PCR of GAPDH
GAPDH-QR	GAAACGACCTGATCCTCGGTGTAG	Real-time PCR of GAPDH
Luc-QF	GCTGGTGCCAACCCTATTCTCC	Real-time PCR of Luc
Luc-QR	AACCGCTTCCCCGACTTCCTTA	Real-time PCR of Luc
CD63-OF	ATGGGTTTTGTTTCGACACTCGCCAG	ORF check for Pu-CD63
CD63-OR	CTAAAAGTCTTTATGACCTGTCTTG	ORF check for Pu-CD63
RiCD63-F	TAATACGACTCACTATAGGGAGAGGGCTTGTTTATTCGCTTCGGGTCA	RNAi primer for CD63
RiCD63-R	TAATACGACTCACTATAGGGAGATAGTTGTTTGCTGTCGTCGCTGTGG	RNAi primer for CD63
RiLuc-F	TAATACGACTCACTATAGGGAGAGTCCGTTCGGTTGGCAGAAGCTATG	RNAi primer for Luc
RiLuc-R	TAATACGACTCACTATAGGGAGACATGCGAGAATCTCACGCAGGCAGT	RNAi primer for Luc

“F” indicates the forward primer; “R” indicates the reverse primer.
