# Supplementary material for: CD63 Promotes Hemocyte-Mediated Phagocytosis in the Clam, Paphia undulata
Source: J Immunol Res. 2016 Oct 27;2016:7893490. doi: 10.1155/2016/7893490 (PMC5102739; doi:10.1155/2016/7893490)
Supplement: Supplementary file 1 — Supplementary Figure 1: The complete cDNA and deduced amino acid sequence of Pu-CD63. Supplementary Figure 2: Phylogenetic tree of CD63. [file 7893490.f1.docx]

169 atgggttttgtttcgacactcgccagaatttttctggtggttgtg

M G F V S T L A R I F L V V V

214 aatgttttatttctgattctgggcctcgtgtttttcggactgggc

N V L F L I L G L V F F G L G

259 ttgtttattcgcttcgggtcatctatcttgaataaatacgtggag

L F I R F G S S I L N K Y V E

304 aacgtgaaggattcaattgagcagtcgtcgggaagtgctgggttt

N V K D S I E Q S S G S A G F

349 ggtacagtggatctttctagttttgatataaccgatctgttgtct

G T V D L S S F D I T D L L S

394 ggagtagcccttggactgattttctttggcctgtttctgaccatc

G V A L G L I F F G L F L T I

439 atcagcgtgcttggatgctgcggaggatgctgcaagattaaatgt

I S V L G C C G G C C K I K C

484 atgctcatcacgtatgtgattatttgtatagtattattcctatgt

M L I T Y V I I C I V L F L C

529 caagtggttattattattattctctacggatttccggacacgttc

Q V V I I I I L Y G F P D T F

574 cacaacccagcaaagaagaaacttaaagaagacatacagtcgagt

H N P A K K K L K E D I Q S S

619 tatgtcggtcttaacgggacagacttgaaaacaattagctggaac

Y V G L N G T D L K T I S W N

664 attgttatgcaacaggtgaagtgttgtggtgttgacagttatgct

I V M Q Q V K C C G V D S Y A

709 gattttaccggggcatctaaatggacaacagtttacaattcttac

D F T G A S K W T T V Y N S Y

754 actttaaagacaccgctggcatgctgtaaaagtttacccgattct

T L K T P L A C C K S L P D S

799 acagacttttcttgcgcagacacttccacagcgacgacagcaaac

T D F S C A D T S T A T T A N

844 aactatttagataagggatgttacgacatcatctgggacgccacc

N Y L D K G C Y D I I W D A T

889 cttggaagtactgtcataatggtggcaactctcgttggaatcggc

L G S T V I M V A T L V G I G

934 gtattccagttgctactgattttgtttgggattgtaattttgtgt

V F Q L L L I L F G I V I L C

979 agcatgaagaagaacaagacaggtcataaagacttttag 1017

S M K K N K T G H K D F *

Supplementary Fig. 1 Sequence of Pu-CD63

In blue, conserved CCG motif; in orange, YXXØ motif; in red, putative N-glycosylation sites


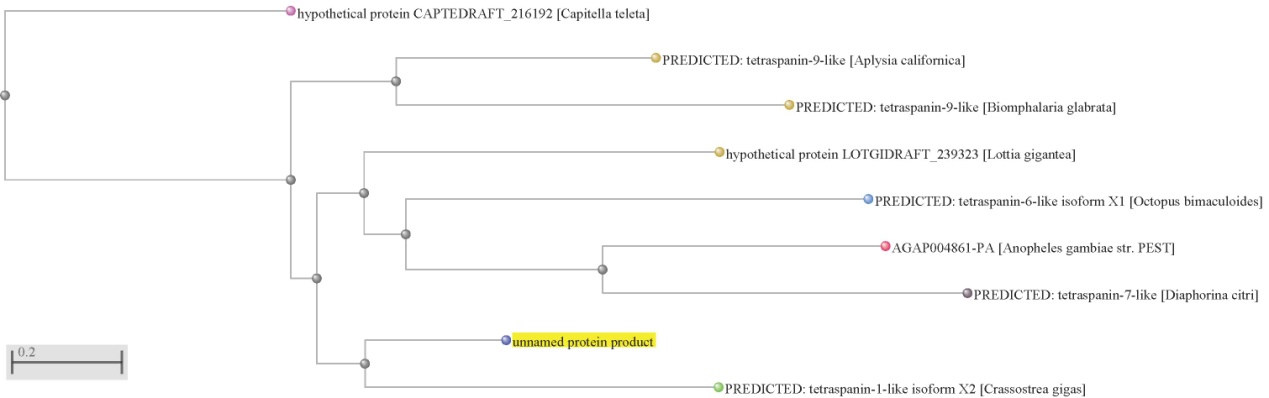


Pu-CD63

Supplement Fig. 2 Phylogenetic relationship of CD63 homolog protein
